# Association between otitis media infection and failed hearing screenings in children

**DOI:** 10.1371/journal.pone.0212777

**Published:** 2019-02-22

**Authors:** Hadara L. Norowitz, Timothy Morello, Hadassah M. Kupfer, Stephan A. Kohlhoff, Tamar A. Smith-Norowitz

**Affiliations:** Department of Pediatrics, State University of New York Downstate Medical Center, Brooklyn, New York, United States of America; University of Ottawa, CANADA

## Abstract

This study aims to assess prospectively whether there is an association between frequencies of upper respiratory tract infections (URTI) or asthma in early childhood and failed otoacoustic emission (OAE) screenings later in life. There are no clear recommendations for hearing testing following acute otitis media (AOM) infection. This is a retrospective, practice based chart review. Participants from a primary care setting were 517 pre-adolescent and adolescent children (49.9% female) (ages 10–21; mean, 15 y/o), who had presented with at least one specific bacterial URTI (AOM, Group A Streptococcus (GAS) tonsillitis, or Influenza) during childhood. Hearing testing was recorded incidentally at all subsequent routine health care maintenance visits (OAE hearing screen). Simple linear regression analyses were performed using R (v3.4.4). We found that number of episodes of AOM infections strongly correlated with number of failed OAE screenings later in life (F = 76.37; *P* = <0.001; R^2^ = 0.1279), while GAS (F = 1.859; *P* = 0.1733; R^2^ = 0.0016) or Influenza infection (F = 2.624; *P* = 0.1059; R^2^ = 0.0031) were not associated with failed OAE screening. Correlation between number of AOM infections and number of failed OAE screenings was not strengthened by presence of asthma. This study found evidence of an association between childhood history of AOM and failed OAE screenings in adolescence. Since this population may be at a higher risk for developing permanent or fluctuating hearing losses, further studies to clarify indications and timing of standard audiological testing among these children should be considered.

## Introduction

Upper respiratory tract infections (URTI) (acute otitis media (AOM), group A streptococcus (GAS), acute pharyngitis and tonsillitis) are common childhood conditions [1] that have been linked to complications including otological changes, and development of atopic disease [2]. Pathogens responsible for these infections include bacterial (*Streptococcus pneumoniae*, *Haemophilus influenzae* and others) or viral (respiratory syncytial virus (RSV), rhinovirus, influenza, adenovirus, and others) [3–6]. However, also of interest but less well investigated, is the effect of other respiratory infections on failed otoacoustic emission (OAE) screenings later in life. This prognosis is important for future audiological diagnoses or intervention.

Krakau, *et al*. studied long-term hearing outcomes and middle ear pathology in a 30-year follow-up in individuals with onset of recurrent AOM (rAOM) before the age of three [7]. They reported that adults who suffered from rAOM as infants did not show any significant hearing loss on standard audiometry [7]. However, there was a trend for impaired results regarding extended high frequency [7]. In contrast to the aforementioned studies, others have reported in a cohort study with 30-year follow-up of hearing, that chronic suppurative OM and rAOM in childhood are associated with adult hearing loss and elevated pure tone thresholds [8].

Possible mechanisms by which AOM can cause direct hearing loss may include pathogens or inflammatory immune responses, which damage the middle ear [3–6]. Structurally, repeated tympanic membrane rupture during episodes of AOM can lead to tympanosclerosis; extended periods of OME can cause thinning of the tympanic membrane. These scenarios typically yield audiometric changes such as mild low frequency conductive hearing loss or ultra-high frequency hearing loss. In more severe cases, COM can cause cholesteatoma, erosion of the ossicles and/or mastoid cavity, all of which can produce a significant conductive or mixed hearing loss.

Studies have shown that there is no long-term impact of otitis media with effusion (OME) on language development in children [9–10]. However, in those studies, the presence, level of severity or timing of hearing loss was not evaluated [10]. Audiological evaluations are often discontinued on asymptomatic children once language has developed and AOM has resolved. Thus, hearing status in late childhood is highly understudied and poses a gap in the literature.

OAE testing is a helpful diagnostic tool which is gaining higher adoption rates in the primary care setting for objectively assessing hearing status [11]. A stimulus is delivered into the ear, which evokes an automatic response (the “otoacoustic emission”) from the cochlea of a healthy ear, which is then recorded and analyzed by frequency. The intact delivery of this recording across the frequency spectrum of audibility relies on the integrity of the whole ear. When coupled with tympanometry, OAE patterns enable a differential diagnosis on temporary hearing conditions versus permanent hearing conditions [11]. OAE also gives insight on severity of middle ear pathology when present. Absent or reduced OAE response does not always mean that a clinical hearing loss is present, but it is grounds for documentation and/or closer audiological monitoring, as these children may be at a higher risk for developing hearing loss. Potential etiologies of an absent or reduced OAE may include physical changes to the outer and middle ear pathway, which attenuate emissions from an otherwise healthy cochlea, or early cochlear outer hair cell dysfunction, which is a harbinger of audiometric sensorineural hearing loss. This early stage of abnormal OAEs, in the absence of clinical hearing loss is known as “hidden hearing loss” [12]; a person may experience subtle changes in hearing beyond pure tone recognition, such as difficulties hearing in environments with noise [12].

When considered among the middle school and high school aged population, a persistently obliterated OAE response assumed to be due to middle ear conditions may overshadow permanent noise-induced hearing changes. These changes are fully preventable and highly topical when considering health risks for this age bracket. Therefore, it is important to understand the long-term otological and audiological changes that may persist in the ears of children with strong early histories of various URTIs.

In the current study, we compared the OAE outcomes in pre adolescent and adolescent children with AOM, GAS, or influenza infections and asthma using data from electronic medical records. This investigation addresses a gap in the literature, which is important for understanding URTIs in early childhood and their possible sequelae including hearing loss.

## Materials and methods

### Participants

We used data obtained from an electronic medical record (EMR) database from an outpatient pediatric private practice (Brooklyn, NY, USA) between 2003–2018. For this retrospective chart study, all patients were older than 10 years at the time of this study, and had at least one lifetime episode of presumed bacterial URTI (AOM, tonsillar pharyngitis caused by group A beta-hemolytic streptococcus). Inclusion criteria also included serious viral URTI such as influenza A or B. The SUNY Downstate Medical Center Institutional Review Board approved this study without the need for written informed consent due to lack of patient identifiers.

### Diagnosis of AOM, GAS or Influenza A: Definitions

Physician diagnosis of unilateral or bilateral AOM was based on symptomatology and determined by otoscopy as recommended by the American Academy of Pediatrics guidelines described in Leiberthal, *et al* [13]. Symptoms in older children with AOM include a history of rapid onset of ear pain. However, in young children, symptoms may include tugging or holding the ear, excessive crying, fever, or changes in the child’s sleep or behavior pattern [13]. Physician diagnosis of GAS in the presence of typical clinical signs and symptoms (sudden onset of fever, sore throat, painful swallowing) was determined by a positive rapid strep test (RST) (Henry Schein One Step Plus Strep A Dipstick Test Kit; Melville, NY) or positive overnight culture (blood agar plate, 5% sheep blood in tryptic soy agar (TSA) base; Thomas Scientific, Swedesboro, NJ). Physician diagnosis of Influenza was defined as a positive Influenza A or B test (OSOM Influenza A & B Test; Sekisui, LLC, San Diego, CA) in the presence of an influenza-like illness (ILI).

### Audiological testing protocol

Audiological testing was administered using a Distortion Product Otoacoustic Emissions hearing screener (Welch Allyn, Skaneateles, NY), which was performed on all patients at routine health care maintenance visits. The frequency of OAE measurements was between 2000 and 5000 Hz. Abnormal hearing result was defined as absence of otoacoustic emissions at 2/6 frequencies between 1000 and 8000 Hz, with a pass/fail result recorded. Patients with a failed screen were ultimately referred to an audiologist for comprehensive evaluation.

### Statistical analyses

The association between AOM, GAS or influenza and failed hearing screenings later in life was modeled using binomial logistic regression and linear regression, with baseline characteristics at study entry. The effect of variable asthma status was determined by linear regression. Adjusted odds ratios are presented with 95% confidence intervals. Mann-Whitney U-tests were performed to calculate *P* values for differences in male and female patients (i.e. failed hearing screen). A two-sided *P* value of < 0.05 was considered statistically significant. All statistical analyses were performed in R using R-Studio (version 3.4.4).

## Results

### Cohort demographic and clinical characteristics

Table 1 provides demographics and characteristics of the study population (N = 517) (50.1% male; ages 10–21, mean 14 y/o) (49.9% female; ages 10–21, mean 15y/o). At study entry, 517 children were included in this analysis. We report 49.9% female; range 10–21; mean, 15±3.71 y/o (median 14.0) and 50.1% males; range 10–21; mean 15± 4.09 y/o (median 15.0). No significant group differences were detected between the male and female patients for number of failed hearing screenings (15% ± 22 vs. 14%± 23; *P* = 0.16). Of the patients, we report asthma (4.1%), atopic dermatitis (AD) (15.3%) and both asthma and AD (1.5%) (Table 1).

**Table 1:** Demographics and clinical characteristics of study population.

Characteristic	Count	Total (%)
**Sex (M/F)**		
Male*	259	50.1
Female**	258	49.9
**Age (y)**		
10–13	227	43.9
14–17	178	34.4
18–21	112	21.7
**Race**		
Asian	3	0.6
Black or African American	4	0.8
White	507	98.1
Unknown	3	0.6
**Condition**		
Asthma only	21	4.1
Atopic dermatitis only	79	15.3
Asthma and Atopic dermatitis	8	1.5
** **	**Males** **(%±SD)**	**Females** **(%±SD)**
**Outcome**		
Failed Hearing Screens†	15±22	14±23

Total N = 517

* Range 10–21 y; mean 15±3.71, median: 14

** Range 10–21 y; mean 15±4.09, median: 15

† P = 0.164

### Association between URTI and number of failed hearing screenings

**Fig 1:**
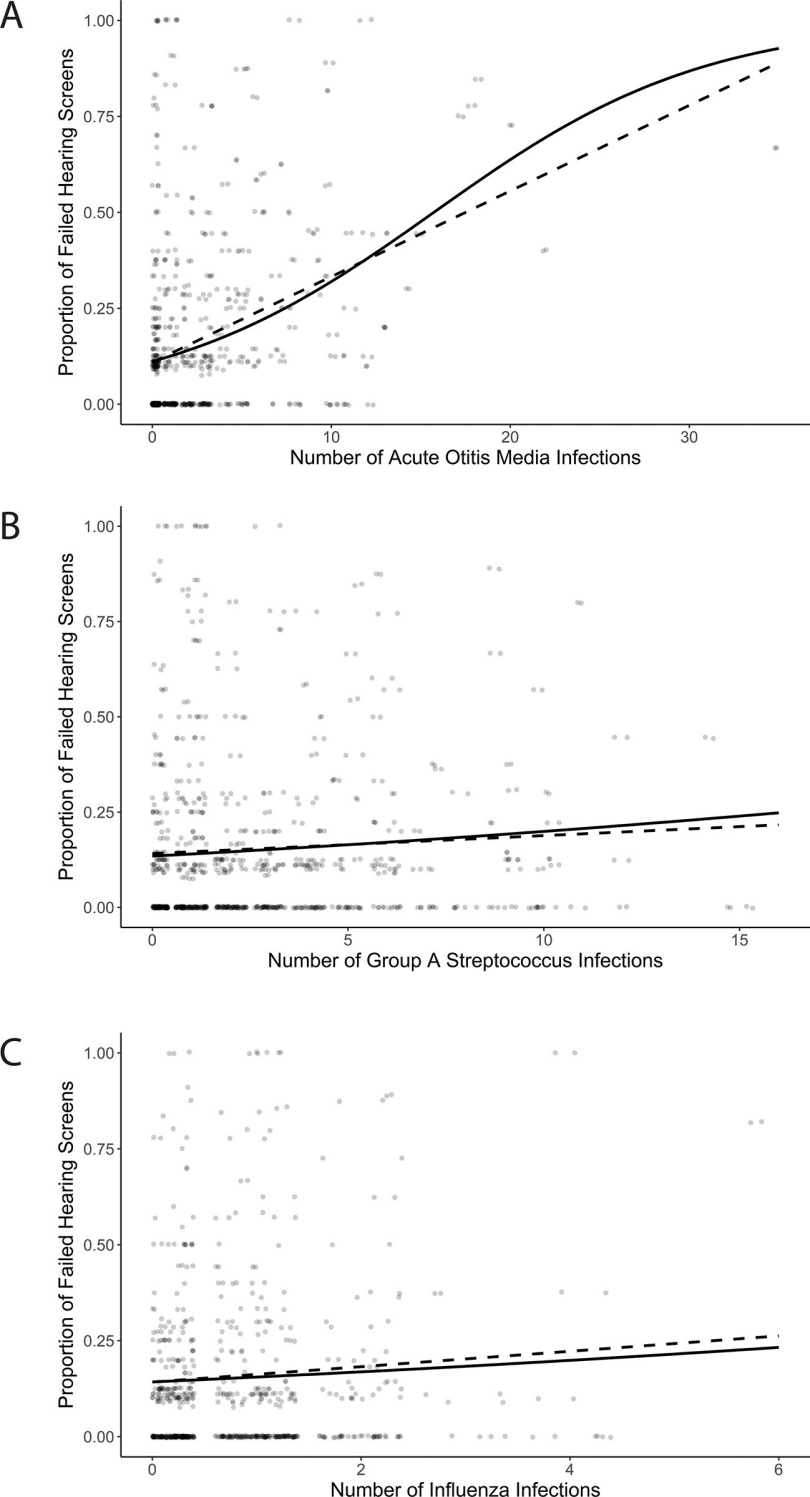
Association between URTI and number of failed hearing screenings. Fig 1A: Number of AOM infections and proportion of failed hearing screenings later in life. Fig 1B: Number of GAS infections and proportion of failed hearing screenings. Fig 1C: Number of Influenza infections and proportion of failed hearing screenings. Proportion of failed hearing screens was calculated as follows: number of failed hearing screens/total hearing screens. Solid line: binomial logistic regression. Dotted line: linear regression.

In our total population, number of episodes of AOM infections strongly correlated with number of failed otoacoustic emission screenings later in life (F = 76.37; *P* = <0.001; R^2^ = 0.1279) (Fig 1A), while number of GAS (F = 1.859; *P* = 0.1733; R^2^ = 0.0016) (Fig 1B) or number of Influenza infection (F = 2.624; *P* = 0.1059; R^2^ = 0.0031) (Fig 1C) were not associated with failed OAE screenings later in life. A repeat analysis of AOM versus failed OAE screenings with the exclusion of 22 patients who had greater than 10 AOM infections also showed a significant correlation (F = 42.62, P = <0.001, R^2^ = 0.07785).

### Association between URTI and number of failed hearing screenings in asthma

**Fig 2:**
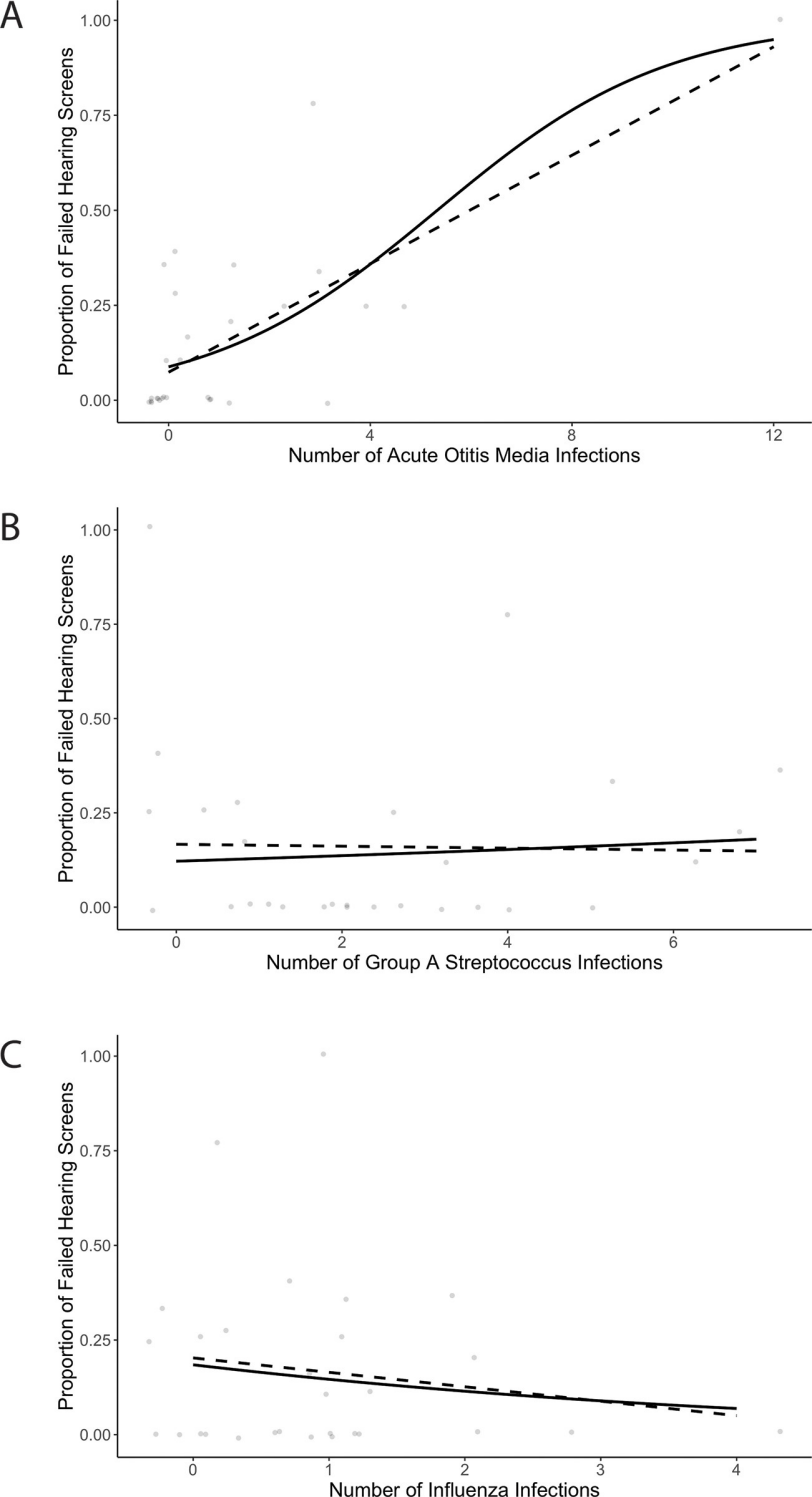
Association between URTI and number of failed hearing screenings in asthma. Number of AOM infections (Fig 1B), Number of GAS infections (Fig 2B), Number of Influenza infections in patients with asthma. Proportion of failed hearing screens was calculated as follows: number of failed hearing screens/total hearing screens. Solid line: binomial logistic regression. Dotted line: linear regression.

In patients with asthma, number of episodes of AOM infections correlated with number of failed OAE screenings later in life (F = 34.04; *P* = <0.0001; R^2^ = 0.5188) (Fig 2A), while number of GAS (F = 0.004159; *P* = 0.949; R^2^ = 0.0014) (Fig 2B), or number of Influenza infection (F = 0.3306; *P* = 0.5696; R^2^ = 0.019) (Fig 2C) infections were not associated with failed OAE screening later in life. All associations reported above were similar in patients with asthma and those without asthma (not shown). The correlation between number of AOM infections and number of failed hearing screenings was not affected or strengthened by presence of asthma diagnosis.

### Age range of AOM infections

**Fig 3:**
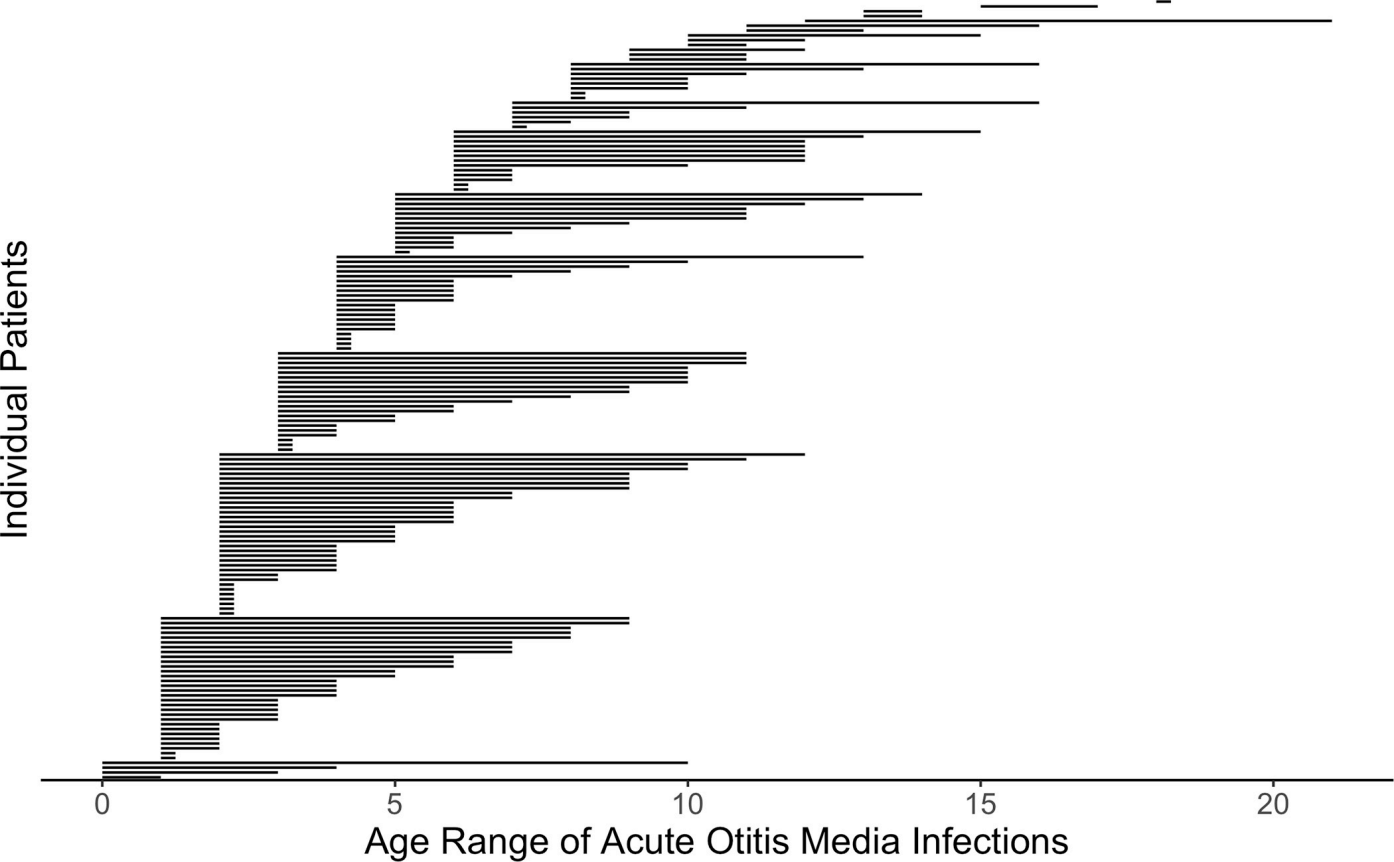
Age range of AOM infections. Each line represents an individual patient. N = 162 represents all patients who had at least one AOM infection.

**Fig 4:**
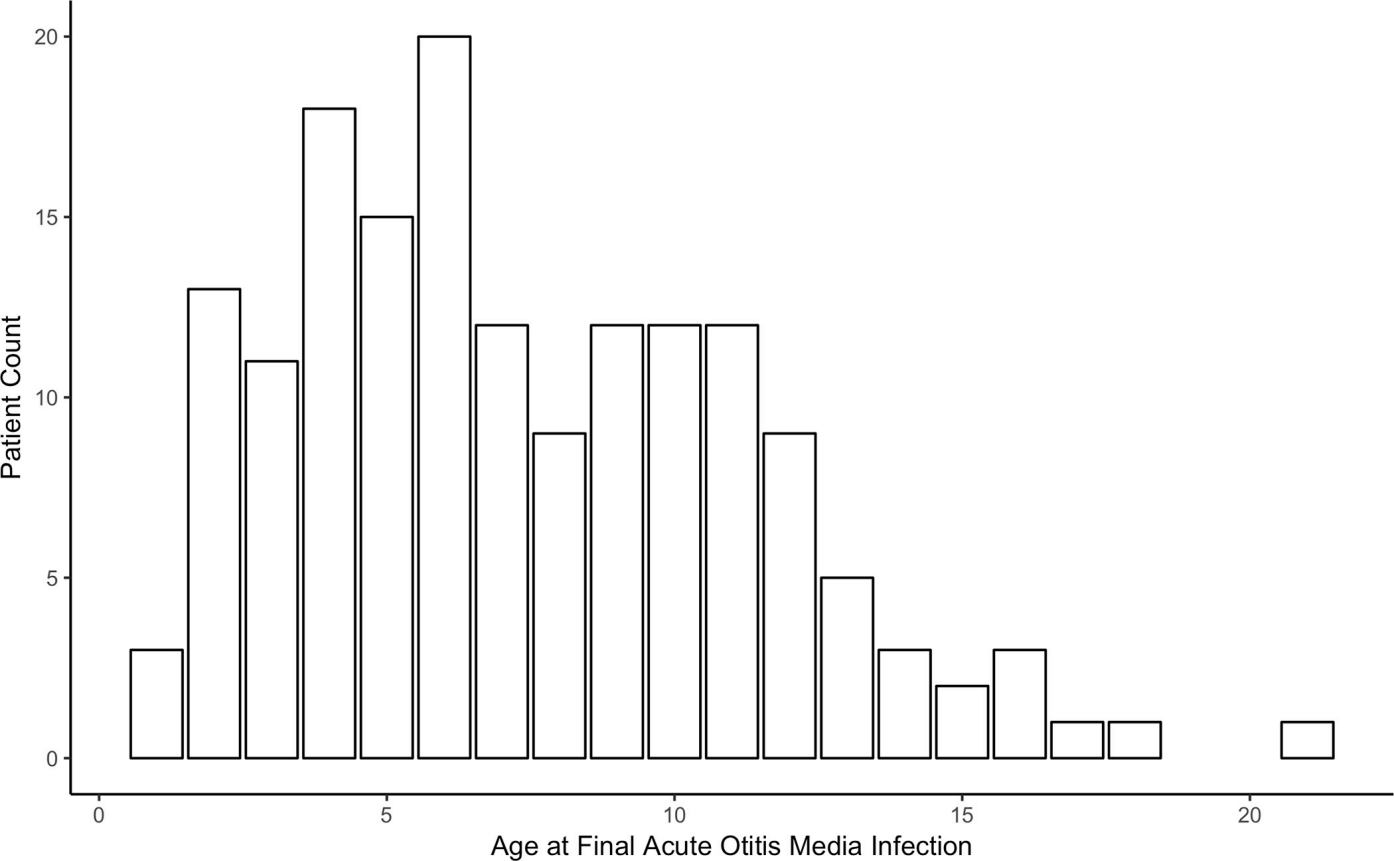
Age at final AOM infection. Histogram showing counts of patient ages at final AOM infection (N = 162).

Age range of AOM infections is represented in Fig 3. By the age of eight >50% of patients who have had at least one AOM infection, have had their final case of AOM (Fig 4).

## Discussion

In the present work, we examine URTIs in early life and sequelae later in life. This is illustrated by a significant association between numbers of AOM episodes in early-life and failed hearing screenings via OAE later in life. Interestingly, there was no independent effect of asthma status. Our study could not reveal significant differences in the numbers of GAS or Influenza infections and failed OAE screenings. The combination of these findings suggests that specifically AOM early in life may contribute to persistent abnormal hearing results in certain patients, even through adolescence. This underlines the importance of closer monitoring for relapse of temporary hearing loss or development of permanent hearing loss, in order to ensure accurate diagnosis and treatment in this at-risk population.

AOM is characterized by otoscopic findings and mostly causes conductive hearing loss by damaging the middle ear [8]. Our main finding was that numbers of AOM episodes, but not GAS or influenza, in early-life, was associated with failed OAE screenings later in life. These results are in agreement with the findings of previous studies based on data that showed that chronic otitis media (COM) and recurrent AOM in childhood are associated with adult hearing loss [8]. The authors concluded that ears with a subsequent hearing loss after OM in childhood age at a faster rate than those without a hearing loss [8]. Lou and Feng [14] reported that the rate of sensorineural deafness caused by OME is higher than expected [14]. However, others have reported that adults, who suffered from recurrent AOM as infants, did not show any clinically significant hearing loss when compared with controls [7]. We speculate that the observed discrepancies in our study could be explained by selection method and high inclusion rate of all subjects in a large pediatric practice. In addition, it could be that some of the hearing loss observed in this study (during adolescence) may not have persisted through adulthood because the oldest patient age followed in this study was up to 21 years. In conclusion, it is plausible to speculate that the damage caused to the outer, middle, or inner ear by multiple AOM infections may affect future hearing loss, and does reveal a true biological occurrence.

The role of URTIs in the context of hearing loss has been theorized in the literature. AOM pathogenesis can involve interplay between virus, bacteria, and the host immune response [15]. It has been reported that viruses can be found in the middle-ear fluid either alone or with bacteria [15]. Viruses can enhance inflammation in the middle ear, and impair the resolution of AOM infection [15]. Vaccination against viruses can prevent viral infections that cause OM [14]; early treatment with antiviral agents can reduce AOM [15]. It should be mentioned that specific pathogens were not tested in our study and diagnosis was based on clinical criteria. Therefore, we cannot attribute effects of particular groups of pathogens to long-term damaged hearing loss, but it would be interesting to explore such interactions in future studies.

Other explanations by which AOM can directly cause hearing loss include that fact that subjects who have had more frequent AOM are more likely to have effusion at the time of the hearing tests (because they may be at higher risk of recent OM, even as teenagers). Current effusion would explain a degree of hearing loss. Lastly, rAOM is associated with an increased risk of chronic otitis media with effusion (COME). Both rAOM and COME are often treated with tympanostomy tube placement (TTP) [16]. This surgery can leave scarring on the tympanic membrane that may cause hearing loss.

Prior literature has reported that repeated infections during childhood are linked to the development of asthma or atopic disease [17–18]. Studies have suggested an association between asthma and recurrent ear infections in young children [19]. Eldeirawi, *et al*. demonstrated that history of OM in children aged 2 to 11 years was associated with history of asthma and wheezing [20]. However, others have demonstrated an inverse relationship between infections during early childhood and risk of subsequent asthma or atopy in children (hygiene hypothesis) [21–22]. These infections may have a protective effect; however, these studies remain inconclusive [20, 23]. Risk factors for early childhood infections include daycare attendance, large household size, or having one or more sibling [23–24]; however, these factors can reduce the risk of asthma and development of atopy [23–24].

The second important finding reported was that diagnosis of asthma did not have any significant effect on the association observed between numbers of AOM and failed hearing screens. Even though previous studies have demonstrated a positive association between asthma and recurrent ear infections in children [19–25], it is uncertain whether the asthma predisposed children to ear infections or whether many ear infections altered the immune response to cause asthma [20]. Allergic rhinitis is a common co-morbidity of asthma, leading to inflammation of the nasopharynx and obstruction of the Eustachian tube [26]. In addition, patients with asthma may have uneven ventilation or bronchospasms, which may decrease arterial oxygen saturation, leading to hypoxemia [27]. Studies have demonstrated that diseases causing hypoxia (i.e. vascular disease), can also induce hearing loss [28].

Even though the American Academy of Pediatrics (AAP) published recommendations for hearing screening beyond the newborn period, pediatricians have little guidance on screening and/or referral criteria past the infant stage [29–30]. The AAP encourages newborn hearing screening and periodic screening for all children through adolescence [31]. Clinically, this recommendation is often overlooked. This may be due to time or instrumentation constraints, or faulty assumptions of normal hearing due to lack of complaints. It should be mentioned that normal hearing for children is considered to be pure tone auditory thresholds at or below 15-dB, and screening failure is defined as the inability to detect one or more frequencies at 20-dB in either ear [30–31]. The AAP recommends audiologic referral for children who fail screening at 25-dB or higher, as well as discussion with the parent of the importance of further evaluation [30–31]. Our results suggest that a higher number of episodes of AOM can be a risk factor for abnormal hearing findings, even once the peak incidence of their upper respiratory tract and middle ear pathologies has passed.

This study has several limitations that need consideration. First, patients in this study diagnosed with AOM do not have a specific microbiologic diagnosis. Second, we do not report non-infectious disease related diagnoses, as these are not consistently documented for all patients in the study population. Third, the distortion-product OAE method used for hearing screening finely detects abnormalities of the auditory pathway which do not always translate into clinical hearing loss. It should also be mentioned that the lack of association between numbers of AOM, GAS, or Influenza infections and number of failed OAE screens in asthma may be due to the small sample size. However, our study has several strengths, including the inclusion of a pediatric cohort from a large single practice with excellent follow-up from birth to adulthood in the same children. This allows a complete chart review analysis in order to ascertain exact numbers of lifetime AOM and other respiratory infections based on clinician assessment and not patient recall, therefore, avoiding differential and non-differential recall bias.

It should be stressed that middle ear pathology can obscure the presence or absence of a permanent hearing issue, which is why it is advisable to preventatively screen later in life once peak incidence of AOM has passed (Fig 4). There is a high rate of false positive failed hearing screenings during youth due to temporary hearing loss/conductive pathologies, which is why physicians tend to question their utility and skip audiological testing. For this reason, OAE screening is a useful test for the adolescent population in the primary care setting.

## Conclusions

AOM is a public health concern with significant financial burdens on the healthcare system [32–33]. Our findings suggest that an abnormal OAE response can be predicted years after OM has resolved. Thus, this disease may have a longer lasting effect on otologic and auditory status than previously thought.

The data presented in this study show that there exists an association between AOM infections in children early in life and failed OAE screenings later in life. This finding has potential implications, in terms of understanding the relationship between AOM and subsequent permanent hearing loss, as well as developing new universal screening approaches to better identify individuals at risk for hearing management and audiological intervention. Since noise-induced hearing loss is a growing public health concern in the adolescent population, it is important that key clinical features arising from early history of OM do not mask new, preventable changes to hearing. Future studies should prospectively evaluate such public health approaches and the impact on hearing loss.
